# Spatio-temporal Organization During Ventricular Fibrillation in the Human Heart

**DOI:** 10.1007/s10439-018-2007-9

**Published:** 2018-03-15

**Authors:** Jinny Robson, Parham Aram, Martyn P. Nash, Chris P. Bradley, Martin Hayward, David J. Paterson, Peter Taggart, Richard H. Clayton, Visakan Kadirkamanathan

**Affiliations:** 1 Department of Automatic Control and Systems Engineering, University of Sheffield, Sheffield, UK; 2 Insigneo Institute for in-silico Medicine and Department of Computer Science, University of Sheffield, Sheffield, UK; 3 Auckland Bioengineering Institute, Auckland, New Zealand; 4 University College London Hospitals, London, UK; 5 University of Oxford, Oxford, UK

**Keywords:** Global myocardial ischaemia, Cross-correlation, Complex network analysis, Hierarchical clustering, Spatio-temporal systems

## Abstract

In this paper, we present a novel approach to quantify the spatio-temporal organization of electrical activation during human ventricular fibrillation (VF). We propose three different methods based on correlation analysis, graph theoretical measures and hierarchical clustering. Using the proposed approach, we quantified the level of spatio-temporal organization during three episodes of VF in ten patients, recorded using multi-electrode epicardial recordings with 30 s coronary perfusion, 150 s global myocardial ischaemia and 30 s reflow. Our findings show a steady decline in spatio-temporal organization from the onset of VF with coronary perfusion. We observed transient increases in spatio-temporal organization during global myocardial ischaemia. However, the decline in spatio-temporal organization continued during reflow. Our results were consistent across all patients, and were consistent with the numbers of phase singularities. Our findings show that the complex spatio-temporal patterns can be studied using complex network analysis.

## Introduction

The heart is an electromechanical pump. During each normal heartbeat an electrical wave of activation is initiated by the heart’s natural pacemaker and spreads through entire heart to both trigger and synchronize mechanical contraction. Any disturbance to the normal pattern of generation and propagation of these electrical activation waves results in an abnormal heart rhythm or cardiac arrhythmia. The most critical cardiac arrhythmia is ventricular fibrillation (VF), during which synchronized and regular contractions of the heart are suppressed by rapid and self-sustaining waves of electrical activation in the ventricles. As a consequence the heart is not able to pump blood effectively and VF is quickly lethal unless halted by defibrillation.^15^

An unavoidable consequence of spontaneous VF is global myocardial ischaemia, resulting from ineffective mechanical function and reduced myocardial perfusion. Several studies in both animal^4^,^14^ and human hearts^3^,^17^ have established that as a result of progressive global myocardial ischaemia, VF exhibits a series of stages where the frequency and the complexity of electrical activation waves vary. Understanding the dynamics of electrical activation waves during episodes of VF with global myocardial ischaemia is of great importance because this knowledge could be used to develop novel therapeutic strategies that could be optimized for each stage of VF.^2^,^24^

Recent technological advances in recording techniques^11^,^21^ along with increasingly detailed models of cardiac electrophysiology^5^ have greatly added to our knowledge of the mechanisms that initiate and sustain VF in the human heart. However, a detailed understanding of these mechanisms remains elusive.

One aspect that is poorly understood is whether the spatio-temporal patterns of electrical activation during VF arise from random or organized processes.^16^ Early studies of VF emphasized the observation of turbulent electrical activation waves.^19^,^30^ In contrast, other studies have found spatial and temporal organization in the pattern of electrical activation.^6^,^13^,^29^ Spatial organization is of particular interest because it could arise from an underlying connectivity structure that drives complex activation patterns on the heart surface. Moreover, in human VF the pattern of electrical activation results from a small number of excitation waves,^29^ compared to animal hearts of similar size.^13^

A key tool in the analysis of activation patterns on the surface of the heart during VF has been phase analysis, which can identify both activation wavefronts and the phase singularities around which they rotate.^7^ Although the complexity of re-entrant activation patterns can be assessed from the numbers and lifetimes of phase singularities,^29^ this approach does not allow any underlying connectivity structure to be identified. The spatial characteristic length based on coherence and correlation functions has been used to quantify the extent of spatial organization in both animal and human VF.^9^ Correlation and coherence functions quantify the degree of functional association between electrical activation waves at different spatial locations in time and frequency domain respectively. These algorithms have shown promise in characterizing the extent of spatial organization in both atrial and ventricular activation sequences particularly in differentiating between normal sinus rhythm and cardiac arrhythmias.^9^,^27^ However, quantifying and monitoring the level of organization during different stages of ischaemic VF in the human heart has not to our knowledge been reported in the literature.

We propose that the level of spatial organization within each episode of VF can be quantified by determining patterns of connectivity underlying electrical activation recorded at different spatial locations. In theoretical neuroscience, analysis of cortical connectivity network, structural, functional and effective, has a long and rich history. Structural connectivity is built upon the anatomy of the brain while the functional and effective connectivity are based on analyzing neuroimaging data.^10^,^28^ In functional connectivity networks, network connections are derived based on correlation or coherence between different regions, whereas network connections in effective connectivity are obtained *via* causal modelling such as Granger causality,^12^ partial directed coherence (PDC)^25^ and more recently model-based frameworks which integrate anatomical information with multi-electrode electrophysiological recordings.^1^ Another important approach is graph theory analysis which explores properties of complex networks and has been extensively applied to brain connectivity data.^28^ Clustering techniques have been also applied to time series data or its corresponding functional network to group regions with similar functional activities.^18^ These techniques can be adopted to study the spatial and temporal activation patterns in the human heart. For example the effective connectivity network based on the PDC approach has been used to analyze intracardiac signals recorded during atrial fibrillation (AF).^25^ However, many of these methods have not yet been applied to human VF, and there is a sufficient volume of interesting results from theoretical neuroscience studies that warrants further investigation in this domain.

The aim of this study was therefore to evaluate techniques to quantify the level of spatial and temporal organization in electrical activation sequences during human VF. First, we used the cross-correlation function to develop a method for quantifying functional association between recordings from each epicardial electrode and hence to compute the global level of spatio-temporal organization. Second, we exploited the spatio-temporal network structure obtained based on cross-correlation coefficients to study the organization of electrical activation sequences as spatio-temporal functional networks. We also applied graph-theoretical measures to further quantify the properties of network-based representation. To examine and visualise the spatio-temporal patterns of similar functional association over different regions of the epicardial surface, a hierarchical clustering method based on the cross-correlation matrix was also employed. Temporal progressions of the underlying connectivity structures obtained from these methods were then quantified and monitored using a sliding window-based analysis.

## Materials and Methods

### Data Acquisition and Preprocessing

The recordings used in this study were from a group of ten patients (labelled as H055, H057–H060, H062–H066) described in a previous publication^3^ in which the patient details and methods used for data acquisition are covered in detail. Briefly, each recording was collected from patients undergoing cardiopulmonary bypass for routine cardiac surgery with cross clamp fibrillation, and the study was approved by the local hospital ethics committee (REC 01/0130).

Epicardial unipolar electrograms were recorded using an elasticated sock consisting of 256 electrodes with an electrode spacing of approximately 10 mm, which was placed over the epicardial surface of the ventricles following cannulation for cardiopulmonary bypass (CPB). Unipolar signals from each electrode were collected with a sampling rate of 1 kHz using a UnEmap system (Auckland UniServices Ltd, New Zealand) with the reference electrode placed on the chest retractors. After the beginning of CPB, VF was induced by 50 Hz AC burst pacing. The electrograms were continuously recorded for a total duration of 3.5 mins. During the first 30 s coronary perfusion was maintained (control VF). After 30 s the aorta was cross-clamped between the coronary sinus and the CPB cannula, and coronary perfusion was interrupted for 150 s (ischaemic VF subdivided into 5 epochs, each 30 s long). The cross clamp was then removed, and a further 30 s of VF electrograms were recorded (reflow VF).

**Figure 1:**
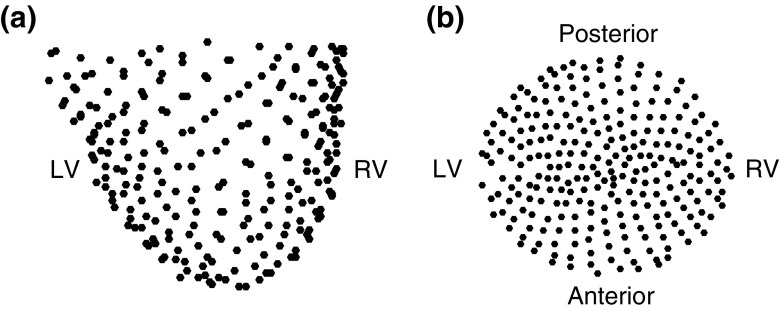
Representation of epicardial sock with the spatial coordinates of the epicardial electrodes (black circles) with respect to left and right ventricles (LV and RV). (a) Three-dimensional view of electrodes; (b) projection of electrodes onto flat two-dimensional surface.

Epicardial electrograms were pre-processed using two major steps. First, slow variations due to respiratory artefacts were removed from each channel with piecewise linear detrending. This also ensured that the mean of each preprocessed fibrillation electogram was approximately zero. The second step was to identify and exclude electrodes with poor quality signals. Only electrograms with dominant frequencies (DF) between 2 and 20 Hz were included in the analysis. Following this a visual examination was further performed to exclude electrograms with amplitudes close to zero. As a result, an average of 219 electrodes (range 186–240) with good quality signals throughout the recording were available for the analysis. The positions of epicardial electrodes in three-dimensional space were obtained by fitting the multi-electrode sock over a heart model. The three-dimensional epicardial electrode coordinates (Fig. 1a) were projected onto a cone-shaped surface enclosing both ventricles. This surface was then projected onto a two-dimensional flat disc (Fig. 1b).

### Linear Coupling Measure

In order to quantify the level of functional association between fibrillation electrograms a linear coupling measure based on cross-correlation functions was employed. Cross-correlation coefficients were obtained from a pre-defined set of time lags and used to calculate the peak magnitude of linear dependency. The range of time lags was derived from the average of local activation cycle lengths over all electrodes. The normalized cross-correlation coefficient between a pair of preprocessed electrograms $y_{i}(t)$ and $y_{j}(t)$ can be calculated by

1 $$\begin{aligned} r_{i,j}(\tau )=\frac{\sum _{t=1}^{N-\tau } y_{i}(t) y_{j}(t+\tau )}{\root \of {\sum _{t=1}^{N}y_{i}(t)^2 }\root \of {\sum _{t=1}^{N}y_{j}(t)^2}} \end{aligned}$$

where N is the number of samples and $\tau \,\in \{-\,d,..0..,d\}$ is the time lag. The total lag, 2*d*, was set to the maximum value of the average activation cycle which we estimated from the average DF of epicardial recordings. The DF of an electrode was defined as the modal frequency in its spectrum calculated using Welch’s power spectral density method with a window length of 3000 samples and 1500 samples overlap.

In order to characterize the overall linear organization we defined a correlation matrix, R, such that

2 $$\begin{aligned} R_{i,j} =r_{i,j}(\arg \max _{\tau }|r_{i,j}(\tau )|) \end{aligned}$$

A single measure of linear organization was then obtained to quantify the overall level of organization using

3 $$\begin{aligned} c=\frac{||R||_F}{n} \end{aligned}$$

where n was the total number of electrodes and $||\cdot ||_F$ denotes the Frobenius norm.

### Complex Network Analysis

In complex network analysis a network is defined as a collection of interrelated elements, which can be represented by a graph. The abstract mathematical formulation of a graph is defined as $G=(V,E)$ where $V=\left\{ V_i\right\}$ is a set of nodes or vertices and $E=\left\{ V_i,V_j \right\}$ is a set of edges representing the connections between the nodes. The edges of a graph structure can be undirected or directed and binary or weighted.

We used a weighted undirected graph with no self or multiple connections is to represent the network structure. The nodes of the network were defined as epicardial electrodes and the weighted edges are the pairwise correlations obtained from the correlation matrix defined in Eq. (2). The edges can be interpreted as functional connectivity, i.e., a symmetrical association between regions of epicardial surface. For further analysis the network structure of weighted graph was represented by its connectivity matrix where rows and columns indicate nodes and matrix elements indicate weights. This is equivalent to the correlation matrix, *R*, with its main diagonal set to zero to exclude self-connections.

In order to explore the connectivity matrix quantitatively we calculated a set of graph-theoretical measures for all nodes (electrodes) using adjacency and weights matrices. The weights matrix was obtained by thresholding the connectivity matrix where entries under a certain value were set to zero. The adjacency matrix was then obtained by replacing non-zero values with one in the weights matrix. It should be noted that thresholding was performed based on the absolute value of correlation coefficient.

Graph theoretical measures provide a powerful tool for a systematic study of the epicardial functional network, providing a quantitative connectivity structure which characterizes the level of organization of fibrillation electrograms over VF episodes. There are several graph-theoretical measures for analysis of complex networks in the literature. In this study connection density, node strength and strength distribution, and mean clustering co-efficient were chosen and calculated using the Brain Connectivity toolbox in Matlab.^28^ A brief description of each measure based on adjacency and weights matrices (undirected and weighted) is provided below.

#### Connection Density

Connection density is defined as the ratio of total number of edges in a network (non-zero entries of adjacency matrix) to the maximum possible number of connections,^28^

4 $$\begin{aligned} \rho = \frac{2n_{{\text{e}}}}{n_{v}(n_{{\text{v}}}-1)}, \quad 0\le \rho \le 1 \end{aligned}$$

where $n_{{\text{v}}}$ is the total number of nodes and $n_{{\text{e}}}$ is the total number of edges in the network.

Connection density can be calculated for a set of adjacency matrices obtained from different threshold values ranging from 0 and 1. A plot of connection density vs. threshold values can be then produced. To calculate the overall level of sparseness or interconnectedness in a network, we calculated area under the curve (AUC) measure that is independent of correlation-threshold value. This measure can be then used to monitor the changes in the overall level of organization across different VF episodes.

#### Node Strength and Strength Distribution

In a weighted graph node strength incorporates information about the total number of connections (degree) and the magnitude of functional association of individual nodes.^28^ The node strength is defined as the sum of weights of all edges incident on a node which can be calculated by

5 $$\begin{aligned} S_{i} = \sum _{j\in {n_{{\text{v}}}}}{a_{ij}w_{ij}} \end{aligned}$$

where $a_{ij}$ and $w_{ij}$ are elements of adjacency and weights matrices respectively.

#### Clustering Coefficient

Clustering co-efficient can be used to examine densely interconnected groups or clusters with similar functional association. For an undirected and binary graph, the local clustering co-efficient of a node was defined as the number of triangles within its neighbourhood (its immediately connected neighbours) to the maximum possible number of edges between them.^28^

We used the algorithm which generalises the clustering coefficient for weighted networks by replacing the number of triangles with the sum of triangle intensities in the neighbourhood of a node.^22^ In this method the local clustering coefficient of a node, *i*, is calculated by

6 $$\begin{aligned} C_{i}=\frac{2l_{i}}{k_{i}(k_{i}-1)} \end{aligned}$$

where $k_{i}$ is the degree of node, *i* and $l_{i}$ is the total intensity of triangles attached to node, *i*.

The intensity of each triangle is defined as geometric mean of its connection weights giving

7 $$\begin{aligned} l_{i}= \sum _{j,h} ({w_{ij}w_{jh}w_{hi}})^{\frac{1}{3}} \end{aligned}$$

Note that weights are normalized by the largest weight in the network. The local clustering coefficients, $C_{i}$, can be then averaged across all nodes to quantify the average clustering coefficient of the whole network, i.e.,

8 $$\begin{aligned} C = \frac{1}{n_{{\text{v}}}}\sum _{i}{C_{i}}, \quad 0\le C \le 1 \end{aligned}$$

Similar to connection density the average clustering coefficients can be calculated for different threshold values. In a plot of average clustering coefficients against threshold values, the AUC measure can be used to quantify the overall interconnectedness in the network.

### Hierarchical Clustering Based on Correlation Matrix

In order to characterize patterns with similar functional association and to quantify the level of spatial organization over the epicardium surface, a hierarchical clustering method based on the correlation coefficients was implemented.^18^
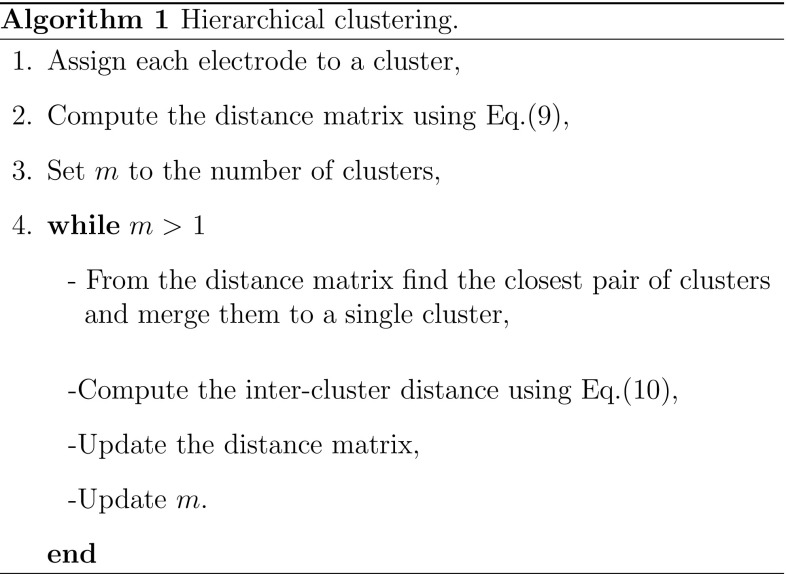


Hierarchical clustering is an agglomerative clustering approach which is initialized by considering each epicardial electrode as a cluster of its own. The first step is to quantify a measure of similarity or dissimilarity based on which clusters of closest functional association are merged to form larger clusters. Here we consider a distance or dissimilarity matrix calculated using the correlation matrix, *R* (Eq. (2)),

9 $$\begin{aligned} D_{i,j}=1-| R_{i,j}| \end{aligned}$$

From Eq. (9) it can be seen that a pair of electrodes with highest correlation coefficient forms a cluster with a distance or dissimilarity close to zero. Once larger clusters are formed the distance matrix should be updated using inter-cluster distances or linkage matrix as newly formed clusters consist of more than one electrode. The most commonly used linkage methods are single, complete and average linkage. Single and complete linkage methods respectively adopt the distance between the closest and farthest neighbouring electrodes to evaluate the inter-cluster distances. These methods do not account for the cluster structure and could be sensitive to outliers. On the other hand average linkage method calculates the average distance between all electrode pairs which makes the method more robust to outliers and hence more accurate compared with single and complete approaches.^8^ We used hierarchical clustering based on average linkage given by

10 $$\begin{aligned} D_{X,Y}=\frac{\sum _{x\in X}^{n_{X}}\sum _{y\in Y}^{n_{Y}} D_{x,y}}{n_{X}n_{Y}} \end{aligned}$$

where $n_{X}$ and $n_{Y}$ are the total number of electrodes in cluster *X* and cluster *Y* respectively.

The hierarchical clustering algorithm based on the correlation matrix is given in Algorithm 1. The result of hierarchical classification can be then represented as a dendrogram. In order to divide the dendrogram into different sub-clusters a cut-off distance measure should be chosen. The resulting clusters below the selected cut-off distance represent spatial regions with similar functional properties. The total number of clusters at different cut-off distances were measured and then normalized with respect to the total number of electrodes. A plot of normalized number of clusters against cut-off distance was then produced. The AUC was then calculated and used to measure the changes in number of clusters over different VF episodes.

**Figure 2:**
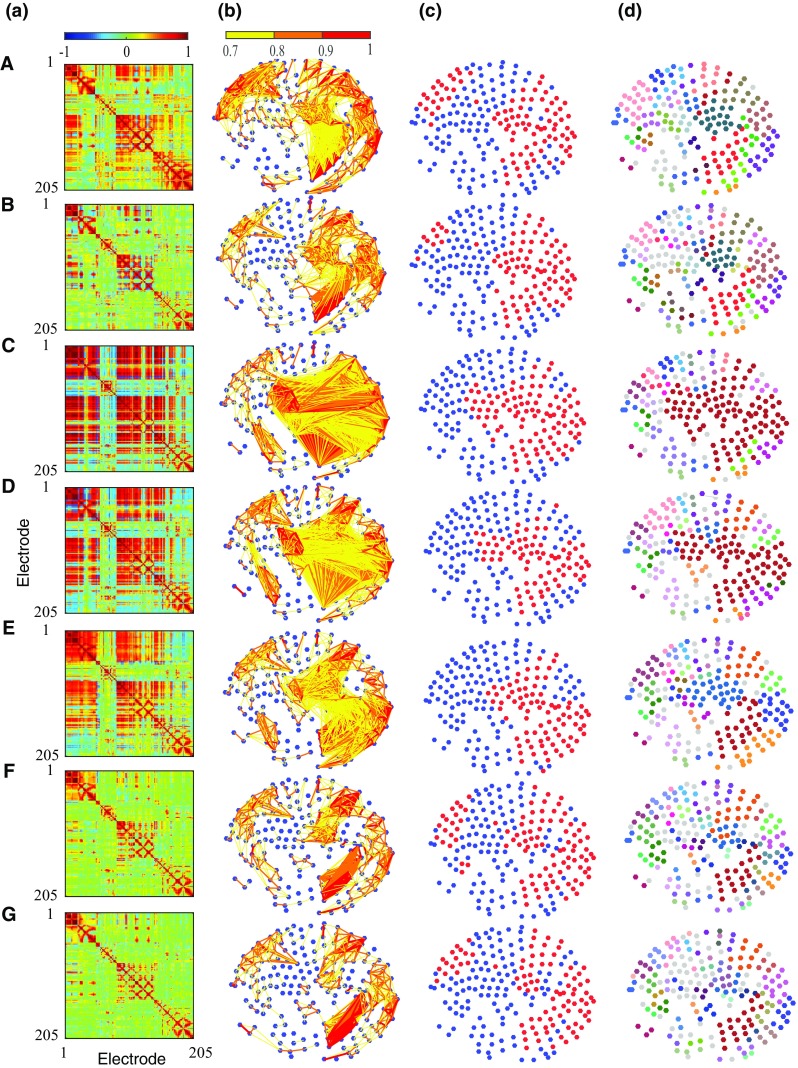
Results from complex network analysis for Patient H055. Each row shows a VF epoch of 30 s duration. (A) controlled VF (Control), (B)–(F) global myocardial ischaemia (Isch1–5), (G) reflow. (a) Correlation matrices; (b) network structure representation with network connections thresholded at value of 0.7; (c) highly correlated nodes from node centrality measure (red circles); (d) clusters from hierarchical clustering; electrodes in same clusters are shown with same colour. Electrodes which do not form a cluster are shown by white circles.

## Results

### Linear Coupling Measure

In order to define the total number of lags in correlation analysis, we calculated the maximum activation cycle length for control VF, global myocardial ischaemic VF and reflow VF separately. Within each VF episode the average value of activation cycle length was estimated as the inverse of mean DF across all electrodes. For each patient this gave a mean activation cycle length for each segment of the time series. The calculated mean activation cycle lengths for ten patients were then averaged for each VF episode. The resultant cycle length during control VF was $180 \pm 19.9$ ms (mean ± 99% confidence interval (CI)). For ischaemic VF this increased from $165 \pm 14.7$ ms to $207 \pm 24.9$ ms and reduced rapidly to $161\pm 22.3$ ms during reflow. Based on the maximum value of activation cycle length the range of time delays was set to $-\,116$ to 116 ms, giving a cycle length of 232 ms. Using the calculated cycle length the correlation matrix for each 30 s VF epoch was computed.

**Figure 3:**
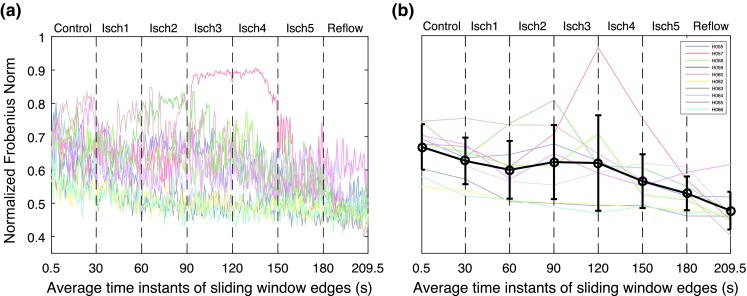
Temporal changes in the quantitative measure of overall organization in correlation matrices during controlled ventricular fibrillation (Control), global myocardial ischaemia (Isch1–5) and reflow across recordings. (a) Normalized Frobenius norm measure; (b) Piece-wise linear fit for the normalized Frobenius norm during VF episodes.

Correlation matrices from the clinical recording of Patient H055 (Fig. 2a) show the presence of functional organization among electrograms in different regions, which changes over the time-course of VF. To monitor temporal changes in the overall level of organization we performed a sliding window-based analysis. The window size was set to 1000 samples (1 s) with an overlap of 500 samples. The overlap was used to reduce the losses in temporal resolution between the window segments. This resulted in 420 window segments for each patient. The overall organization was then quantified by calculating the normalized Frobenius norm of the correlation matrix in each window segment using Eq. (3). The computed Frobenius norms for the clinical recordings are shown in Fig. 3a.

In order to quantify the changes within each VF epoch, we then fitted a piecewise linear model to the computed Frobenius norm from each recording. The break points between linear segments in the model were set as the onset time of 30 s VF epochs. For each patient the parameters of the model were given by an intercept and the change in slope between each VF epoch. Figure 3b shows the piecewise linear fit to the norm in each of the ten patients where black line shows the average fit over ten patients. The corresponding parameters of the mean regression model are given in Table 1. The results from this analysis shows that the mean of Frobenius norm decreases over time-course of VF episodes. During control VF, the mean intercept of Frobenius norm fit was decreased from $0.667 \pm 0.068$ to $0.626 \pm 0.069$ (mean ± SD). During global ischaemia, this measure gradually decreased to $0.529 \pm 0.050$. However there are distinct episodes during global ischaemia in each of the clinical recordings with higher level of organization. In particular, Patient H057 shows a significant jump during the third and the fourth episodes of ischaemia. Highly organized ischaemic VF can be also observed in the second episode for Patient H063 and the beginning of the forth episode for Patient H058. After an increase in the level of organization VF follows a progressive decrease in the level of organization, showing transition to an irregular activation sequence with increased complexity. This transition from higher level of organization to the increased complexity during global ischaemia was observed in all the patients. Subsequently, the mean intercept of Frobenius norm significantly decreased to $0.477 \pm 0.056$ in reflow episode. In all the patients, Frobenius norm is lower during reflow compared to control, indicating that VF in the reperfused heart remains disorganized. The window based analysis is capable of capturing the fine changes at different VF epochs compared to correlation analysis. This is evident in Isch 5 and reflow as shown in Figs. 2F and 2G.

**Table 1:** Piecewise linear fit of organization measures.

Measures	Mean intercept	Mean change in slope (%)						
		Control	Isch1	Isch2	Isch3	Isch4	Isch5	Reflow
Frobenius norm	0.667 ± 0.06	− 0.14 ± 0.13	0.051 ± 0.27	0.166 ± 0.22	− 0.086 ± 0.40	− 0.171 ± 0.61	0.058 ± 0.22	− 0.53 ± 0.20
Connection density	0.646 ± 0.07	− 0.15 ± 0.14	0.058 ± 0.30	0.184 ± 0.25	− 0.097 ± 0.44	− 0.182 ± 0.67	0.065 ± 0.23	− 0.035 ± 0.21
Clustering coeff	0.571 ± 0.08	− 0.16 ± 0.14	0.069 ± 0.33	0.184 ± 0.26	− 0.099 ± 0.45	− 0.203 ± 0.69	0.072 ± 0.261	− 0.068 ± 0.21
Number of clusters	0.133 ± 0.04	0.08 ± 0.08	− 0.029 ± 0.15	− 0.090 ± 0.12	0.037 ± 0.14	0.067 ± 0.23	− 0.006 ± 0.11	0.068 ± 0.09

Presented are mean ± SD

### Complex Network Analysis

Functional interactions between different regions of the epicardial surface can be represented as a network structure by calculating a measure of functional dependency between multi-electrode electrograms. We constructed the underlying functional network on the two dimensional projection of epicardial surface. The connectivity matrix can be obtained by thresholding the correlation matrix using a threshold value between 0 and 1. In this section, we used a threshold value of 0.7 to examine the connectivity structures with higher level of functional association. For each VF epoch, the constructed connectivity network based on the connectivity matrix was then represented as a weighted undirected graph. This is shown for Patient H055 in Fig. 2b. These graphs show the presence of both long and short range connectivities with a high level of organization over the time-course of VF. There are a higher number of connections with a higher level of functional association in ischaemic VF episodes. Moreover, the number of connections in reflow is lower compared to control episode implying the irregular activation patterns in the reperfused heart. The network structures shown in Fig. 2b also highlight the presence of nodes with a high number of connectivities across the epicardium. These nodes can be identified using centrality measures such as node strength. We defined the highly correlated nodes as those with strength greater than the mean strength of the network. We observed a relatively consistent spatial regions (across right ventricles) that exhibit highly organized VF across epicardium (Fig. 2c).

**Figure 4:**
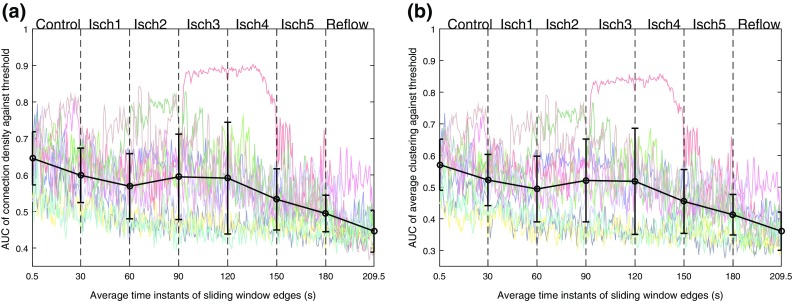
Temporal changes in the quantitative measures from network analysis during controlled ventricular fibrillation (Control), global myocardial ischaemia (Isch1–5) and reflow across recordings. (a) AUC measure of connection density; (b) AUC measure of average clustering co-efficient. Error bar shows the averaged trend from piece-wise linear fit across patients.

We further studied the properties of functional network using a window-based graph-theoretical analysis. The window size and the overlap were chosen similar to the previous analysis. Within each window the connection density (Eq. (4)) and the average clustering coefficient (Eqs. (6)–(8)) were calculated using different threshold values ranging from 0 to 1 with an increment of 0.05. It should be noted that connection density and average clustering co-efficient provide measures of the total number of connections and spatial connectivities in a network respectively. To quantify the overall level of organization at all the threshold values, the AUC of connection density and average clustering coefficient vs. threshold values was then computed. The temporal changes in the AUC of these measures over different VF episodes are shown in Fig. 4. Similarly, we then fitted a piecewise linear model to quantify the trend within each episode and the results from piecewise linear model are shown in Fig. 4 and Table 1. These findings show that both connection density and average clustering coefficient show similar behavior across VF epochs. During control VF, the mean intercept of connection density decreased from $0.646 \pm 0.072$ to $0.599 \pm 0.074$ whereas the mean of average clustering coefficient decreased from $0.571 \pm 0.081$ to $0.522 \pm 0.080$. In global ischaemia, the mean intercepts of the measures decreased to $0.494 \pm 0.050$ and $0.412\pm 0.64$ respectively which was then significantly decreased to $0.44 \pm 0.05$ and $0.36\pm 0.06$ during reflow. This shows that the level of organization quantified using these two measures was low during reflow compared to control VF. This is in accordance with results based on correlation matrix analysis (Fig. 3), indicating that both measures can be used to characterize the global level of organization in VF episodes.

### Hierarchical Clustering Based on Correlation Matrix

In the previous sections we identified nodes with a similar level of functional organization at different locations on the epicardium. We then used a hierarchical clustering approach based on cross-correlation matrix to group nodes into different clusters, to define regions that share similar characteristics. The hierarchical clustering in Algorithm 1 was applied to each of the seven segments VF recording. A dendrogram was then constructed to represent the arrangement of clusters (not shown here). A cut-off distance of 0.3 was then used to split the parent cluster into different sub-clusters. The resulting clusters with $70\%$ similarity in cross-correlation coefficients were mapped onto the two-dimensional epicardium surface (Fig. 2d). This value was chosen to identify the clusters with higher level of functional organization and also to compare with the network structures shown in Fig. 2c. In Fig. 2d a high level of localised spatio-temporal organization can be observed in different regions across VF epochs. As ischaemic VF progresses, larger clusters with a high level of spatial organization were formed (Isch 2, 3 and 4). These clusters were then divided into smaller ones towards the end of the episode (Isch 5). One important observation is the higher number of electrodes which do not form a cluster (white circles) during reflow compared to control.

**Figure 5:**
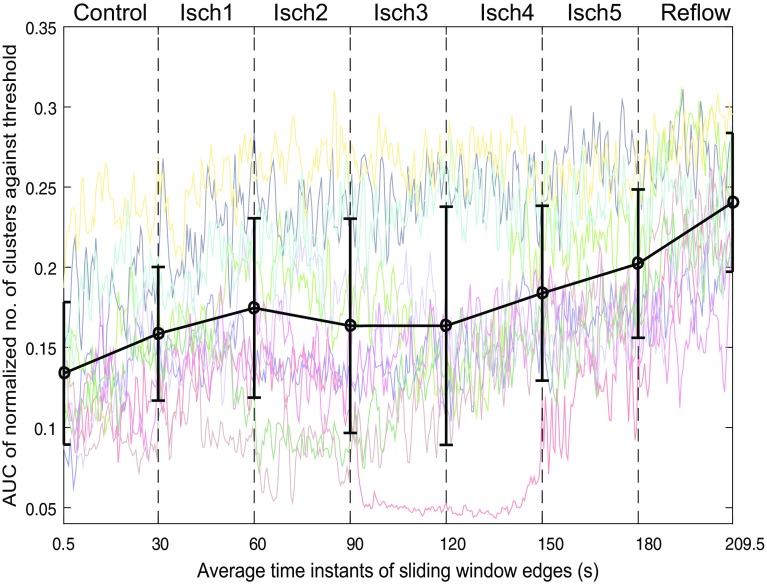
Temporal changes in the AUC of normalized number of clusters during controlled ventricular fibrillation (Control), global myocardial ischaemia (Isch1–5) and reflow across recordings. Error bar shows the averaged trend from piece-wise linear fit across patients.

In order to monitor the changes in the number of clusters over space and time, sliding window-based approach was then employed. The quantity to calculate over each window was the AUC of normalized number of clusters vs. cut-off distances. Similarly, we applied piecewise linear fit to this measure for quantifying the trend within each VF epoch. The results from this analyses are shown in Fig. 5 and Table 1, demonstrating a higher number of clusters in the reflow episode compared to the perfused one. This is consistent with our findings in previous sections as well as with a previous study,^3^ which showed that the level of organization during reflow did not recover to that observed during control. Hence, AUC measure of normalized number of clusters can be used to compare the level of complexity over different VF episodes. Notice the expected opposite trend of this curve to the average clustering coefficient in Fig. 4.

**Figure 6:**
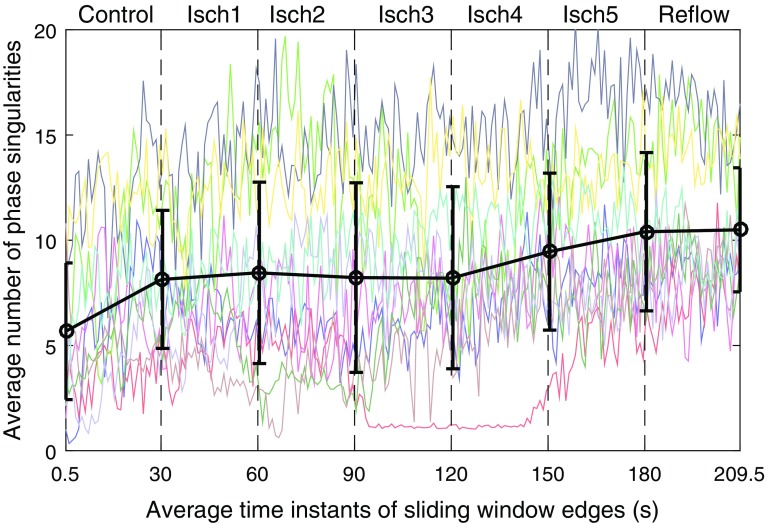
Temporal changes in the average number of phase singularities during controlled ventricular fibrillation (Control), global myocardial ischaemia (Isch1–5) and reflow across recordings.Error bar shows the averaged trend from piece-wise linear fit across patients.

**Table 2:** Comparison of number of clusters and phase singularities.

Patient index	Coefficient of determination
H055	0.60
H057	0.89
H058	0.54
H059	0.62
H060	0.60
H062	0.53
H063	0.67
H064	0.76
H065	0.80
H066	0.60

### Comparison with Phase Analysis

Here, the AUC measure of normalized number of clusters is compared with the number of phase singularities obtained from phase analysis.^3^ Both methods provide measures of the inherent complexity of electrical activation and so should provide consistent results. The temporal changes in the number of phase singularities are shown in Fig. 6. The detailed description of the phase analysis approach used to compute the phase singularities is presented in a previous study.^3^

In order to examine the relationship between the two measures, linear correlation between the two was calculated using coefficient of determination, R-squared, from a linear regression model of the data (Table 2). The R-squared values illustrate that the results obtained from clustering approach is consistent with the phase analysis.

**Figure 7:**
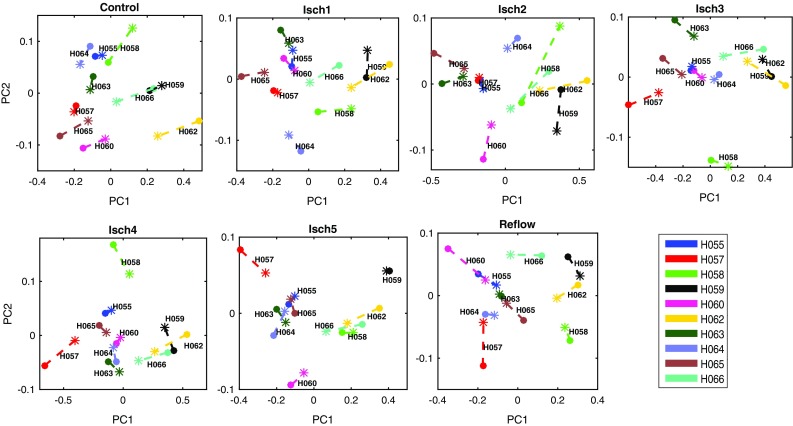
Principal component feature space of complexity measures during controlled VF (control), global myocardial ischaemia (Isch1–5) and reflow. Number of phase singularities (*), number of clusters (•) and difference between the principal components of two quantities are shown in dashed lines.

Temporal changes of the number of clusters and the average number of phase singularities shown in Figs. 5 and 6 also suggest that there are patterns of patients with similar and different complexity levels. We then compared the two complexity measures by examining the clusters of patients determined from principal component analysis (PCA).^20^ Within each 30s VF epoch PCA was applied to AUC measure of normalized number of clusters (Fig. 5) and the average number of phase singularities (Fig. 6). By using the first two principal components, PCA projects the time-varying complexity measures with a dimension of $30\times 10$ samples to $2\times 10$ components in each VF epoch. The resulting PCA feature spaces of the two complexity measures are shown in Fig. 7. It can be seen from the result that the two quantities give identical groups of patients based on level of complexity, while preserving the spatial order.

## Discussion

We have presented a complex network analysis approach to study the spatio-temporal organization in the complex spatio-temporal patterns of human VF. To quantify the level of organization, we have proposed metrics from correlation-analysis, network analysis and hierarchical clustering. Correlation analysis quantified the linear coupling between the epicardial recordings, where the global level of organization within VF episodes was characterized using the Frobenius norm. By representing the functional associations as network connections between the electrodes, the information extracted from network analysis can be used to identify the underlying network of highly interconnected fibrillation regions which drives complex spatio-temporal patterns of electrical activation. Similar to the Frobenius norm measure, connection density provided an overall measure of organization while the spatial organization within a network was characterized using average clustering coefficient. It can be seen that the degree of global clustering is dependent on the global functional organization in electrograms, as the episodes with higher levels of organization show higher levels of clustering and vice versa (Fig. 4). On the other hand, the number of clusters measure from hierarchical analysis characterizes the inherent complexity within VF episodes. Both clustering coefficient and the number of clusters illustrate the presence of localised spatial organization on the epicardial surface through different procedures. Hence the result from one method can be used to examine and validate results obtained from the other approach. The mean change in slope parameters obtained from the piecewise linear fit to proposed metrics (Table 1) show that the changes in organization are consistent throughout the VF episodes. This illustrates that the proposed methods can be used to study the level of organization in VF.

Although the normalized Frobenius norm is an easier approach to estimate the global organization level, an advantage of hierarchical clustering is its ability to detect and visualise clusters of electrodes which share functional properties (Fig. 2d) and to characterize the global complexity level in VF episodes. Moreover the patterns obtained using clustering approach are more robust to outliers as it uses the average linkage method to calculate the inter-cluster distances.^8^

It should be noted that the temporal information, i.e., time delays, between nodes were not investigated in correlation analysis. This is because, cross-correlation function can be sensitive to the inherent noise in the recordings, which significantly affect the estimation of time delays.

Our key findings from this study can be summarized as follows: (1) During control VF, there is a steady decrease in the level of organization; (2) During global ischaemia, there were transient increases within distinct epochs followed by a progressive decrease in the level of organization; (3) During reflow, the level of organization was lower compared to that during perfusion. Our results are in agreement with the findings presented in a previous study,^3^ where the changes in dynamics of complex electrical activation patterns were studied using traditional measures such as phase singularities and dominant frequency. Here we have also demonstrated that the proposed measures of organization are consistent with the number of phase singularities from phase analysis. Phase singularities are identified from the spatial distribution of phase, which requires robust estimation of phase angle from the phase plane trajectory of two state variables. For example, phase plane trajectory can be constructed using voltage and time-delayed voltage as state variables, where an appropriate delay parameter and a stable centre of rotation should be specified.^13^ This method was extended by reconstructing the phase plane trajectory using voltage and its rate of change as state variables.^23^ Although this method does not involve any additional parameters, the inherent differentiation of voltage has the tendency to amplify the noise in the signal. This can be addressed by considering the integral of voltage^26^ or its Hilbert transform^21^ as second state variable. However these approaches involve various pre-processing steps to obtain a stable center of rotation for the robust estimation of phase angle.^7^ The proposed methods in this study do not require these additional pre-processing steps which makes it algorithmically straightforward to implement compared to the phase analysis.

## Conclusion

This paper demonstrates that it is possible to quantify trends in spatio-temporal organization of epicardial electrograms during human VF that could be attributed to coronary perfusion, global myocardial ischaemia and reflow. Our findings show that the complex spatio-temporal patterns can be studied using complex network analysis and hierarchical clustering. These analyses yield results that are consistent with more traditional measures such as the number of phase singularities and dominant frequency, and so provide new tools for quantifying and understanding the dynamics of complex electrical activation patterns in cardiac arrhythmias. One distinct advantage is their simple implementation which do not require additional pre-processing steps. We have found that epicardial activity during human VF becomes progressively more disorganized and complex during a period of global myocardial ischaemia, and that a 30 s period of reperfusion does not reverse this decline.

## References

[1] Aram P, Freestone DR, Cook MJ, Kadirkamanathan V, Grayden DB (2015). Model-based estimation of intra-cortical connectivity using electrophysiological data. NeuroImage.

[2] Barton CW, Cascio WE, Batson DN, Engle CL, Johnson TA (2000). Effect of rates of perfusion on dominant frequency and defibrillation energy in isolated fibrillating hearts. Pacing Clin. Electrophysiol..

[3] Bradley CP, Clayton RH, Nash MP, Mourad A, Hayward M, Paterson DJ, Taggart P (2011). Human ventricular fibrillation during global ischemia and reperfusion clinical perspective. Circ. Arrhythm. Electrophysiol..

[4] Caldwell J, Burton FL, Smith GL, Cobbe SM (2007). Heterogeneity of ventricular fibrillation dominant frequency during global ischemia in isolated rabbit hearts. J. Cardiovasc. Electrophysiol..

[5] Clayton R, Bishop M (2014). Computational models of ventricular arrhythmia mechanisms: recent developments and future prospects. Drug Discov. Today Dis. Models.

[6] Clayton R, Murray A (1999). Linear and non-linear analysis of the surface electrocardiogram during human ventricular fibrillation shows evidence of order in the underlying mechanism. Med. Biol. Eng. Comput..

[7] Clayton RH, Nash MP (2015). Analysis of cardiac fibrillation using phase mapping. Card. Electrophysiol. Clin..

[8] Everitt, B. S., S. Landau, M. Leese, and D. Stahl. Hierarchical clustering. In: Wiley Series in Probability and Statistics, Chichester: Wiley, pp. 71–110, 2011.

[9] Fendelander L, Hsia P-W, Damiano RJ (1997). Spatial coherence: a new method of quantifying myocardial electrical organization using multichannel epicardial electrograms. J. Electrocardiol..

[10] Freestone L, Karoly PJ, Nešić D, Aram P, Cook MJ, Grayden DB (2014). Estimation of effective connectivity via data-driven neural modeling. Front. Neurosci..

[11] Gizzi A, Cherry EM, Gilmour RF, Luther S, Filippi S, Fenton FH (2013). Effects of pacing site and stimulation history on alternans dynamics and the development of complex spatiotemporal patterns in cardiac tissue. Front. Physiol..

[12] Granger CW (1969). Investigating causal relations by econometric models and cross-spectral methods. Econometrica.

[13] Gray RA, Pertsov AM, Jalife J (1998). Spatial and temporal organization during cardiac fibrillation. Nature.

[14] Huizar JF, Warren MD, Shvedko AG, Kalifa J, Moreno J, Mironov S, Jalife J, Zaitsev AV (2007). Three distinct phases of vf during global ischemia in the isolated blood-perfused pig heart. Am. J. Physiol. Heart Circ. Physiol..

[15] Jalife J (2000). Ventricular fibrillation: mechanisms of initiation and maintenance. Annu. Rev. Physiol..

[16] Kaplan DT, Cohen RJ (1990). Is fibrillation chaos?. Circ. Res..

[17] Kazbanov IV, Clayton RH, Nash MP, Bradley CP, Paterson DJ, Hayward MP, Taggart P, Panfilov AV (2014). Effect of global cardiac ischemia on human ventricular fibrillation: insights from a multi-scale mechanistic model of the human heart. PLOS Comput. Biol..

[18] Liu X, Zhu X-H, Qiu P, Chen W (2012). A correlation-matrix-based hierarchical clustering method for functional connectivity analysis. J. Neurosci. Methods.

[19] Moe GK, Harris AS, Wiggers CJ (1941). Analysis of the initiation of fibrillation by electrographic studies. Am. J. Physiol..

[20] Murphy KP (2012). Machine Learning: A Probabilistic Perspective.

[21] Nash MP, Mourad A, Clayton RH, Sutton PM, Bradley CP, Hayward M, Paterson DJ, Taggart P (2006). Evidence for multiple mechanisms in human ventricular fibrillation. Circulation.

[22] Onnela J-P, Saramäki J, Kertész J, Kaski K (2005). Intensity and coherence of motifs in weighted complex networks. Phys. Rev. E.

[23] Packard NH, Crutchfield JP, Farmer JD, Shaw RS (1980). Geometry from a time series. Phys. Rev. Lett..

[24] Plank G, Leon LJ, Kimber S, Vigmond EJ (2005). Defibrillation depends on conductivity fluctuations and the degree of disorganization in reentry patterns. J. Cardiovasc. Electrophysiol..

[25] Richter U, Faes L, Cristoforetti A, Masè M, Ravelli F, Stridh M, Sörnmo L (2011). A novel approach to propagation pattern analysis in intracardiac atrial fibrillation signals. Ann. Biomed. Eng..

[26] Rogers JM (2004). Combined phase singularity and wavefront analysis for optical maps of ventricular fibrillation. IEEE Trans. Biomed. Eng..

[27] Ropella KM, Sahakian AV, Baerman JM, Swiryn S (1989). The coherence spectrum. a quantitative discriminator of fibrillatory and nonfibrillatory cardiac rhythms. Circulation.

[28] Rubinov M, Sporns O (2010). Complex network measures of brain connectivity: uses and interpretations. Neuroimage.

[29] Ten Tusscher KH, Hren R, Panfilov AV (2007). Organization of ventricular fibrillation in the human heart. Circ. Res..

[30] Wiggers CJ (1940). The mechanism and nature of ventricular fibrillation. Am. Heart J..

